# Extended Tenon Removal Versus Limited Tenon Removal With Conjunctivo-Limbal Autograft in Primary Pterygium Surgery: A Prospective Comparative Study

**DOI:** 10.7759/cureus.102128

**Published:** 2026-01-23

**Authors:** Jayant Sharma, Rakesh K Gupta, Shagun Korla

**Affiliations:** 1 Department of Ophthalmology, Maharishi Markandeshwar Medical College and Hospital, Solan, IND

**Keywords:** conjunctivo-limbal autograft, corneal astigmatism, pterygium surgery, recurrence, tenon’s layer excision

## Abstract

Background

Pterygium is a common fibrovascular conjunctival disorder that may cause astigmatism, visual impairment, ocular discomfort, and cosmetic concerns. Surgical excision remains the definitive treatment; however, recurrence continues to be a major challenge. Conjunctivo-limbal autograft transplantation has been widely adopted to reduce recurrence, yet the role of Tenon’s layer excision in improving surgical outcomes remains insufficiently explored. This study was undertaken to compare the outcomes of conjunctivo-limbal autograft transplantation performed with extended Tenon removal versus limited Tenon removal in primary pterygium surgery.

Methods

This prospective comparative study was conducted in the Department of Ophthalmology at a tertiary care center and included 60 patients with Grade 2 or Grade 3 primary nasal pterygium. Patients were alternately allocated into two groups of 30 each. Group A underwent pterygium excision with extended conjunctival and Tenon removal followed by autologous serum-assisted conjunctivo-limbal autograft transplantation, while Group B underwent excision with limited Tenon removal followed by the same grafting technique. Preoperative evaluation included slit-lamp examination, keratometry, and assessment of uncorrected and best-corrected visual acuity. All surgeries were performed by a single surgeon under peribulbar anesthesia. Postoperative follow-up was done at 24 hours, one week, one month, three months, and six months. Outcomes assessed included astigmatism, visual acuity, postoperative complications, and recurrence. Statistical analysis was performed using IBM Corp. Released 2021. IBM SPSS Statistics for Windows, Version 27. Armonk, NY: IBM Corp.

Results

The mean age of the study population was 53.45 ± 8.42 years, with males constituting 43 of 60 patients (71.7%). Grade 2 pterygium was the most common presentation, observed in 59 patients (98.3%). Mean corneal astigmatism significantly reduced from 2.02 ± 1.10 diopters preoperatively to 0.81 ± 0.54 diopters at six months postoperatively (p < 0.05). Early postoperative complications included mild subconjunctival hemorrhage in 45 of 60 patients (75.0%) and graft edema in 9 of 60 patients (15.0%), with no significant difference between the two groups. At six-month follow-up, recurrence was noted in 1 of 30 patients (3.3%) in the extended Tenon removal group and in 4 of 30 patients (13.3%) in the limited Tenon removal group; however, this difference was not statistically significant (p = 0.161).

Conclusion

Conjunctivo-limbal autograft transplantation combined with extended Tenon removal demonstrated a lower recurrence rate and fewer graft-related complications compared to limited Tenon removal, although the difference was not statistically significant. Both surgical approaches resulted in significant improvement in astigmatism and visual acuity. The findings highlight the contributory role of Tenon’s layer in pterygium recurrence and support a comprehensive surgical approach for optimal outcomes. Further studies with larger sample sizes are warranted to substantiate these observations.

## Introduction

Pterygium, derived from the Greek word pterygos, meaning “wing,” is a fibrovascular proliferative disorder of the conjunctiva that extends onto the corneal surface. The majority of pterygia are located nasally, although temporal involvement has also been reported. Pterygium adversely affects vision and ocular aesthetics, and surgical excision is often required in progressive or symptomatic cases. Patients commonly present with ocular redness, foreign body sensation, irritation, itching, watering, restricted ocular movements, and visual disturbances, particularly in advanced disease [1]. Visual impairment primarily results from encroachment onto the visual axis and corneal traction, leading to induced astigmatism [2]. Morphologically, pterygium consists of three components: the cap, head, and body or tail, and chronic cases may demonstrate iron deposition in the basal layer of the corneal epithelium known as Stocker’s line, indicating long-standing disease. Although the exact etiology remains unclear, several studies suggest that prolonged exposure of limbal tissue to ultraviolet (UV-B) radiation results in p53 tumor suppressor gene alterations, leading to abnormal conjunctival proliferation [3].

Multiple mechanisms have been proposed to explain pterygium-induced astigmatism, including pooling of the tear film at the advancing edge of the lesion and mechanical flattening of the cornea caused by fibrovascular traction. Keratometry, corneal topography, and refraction are commonly used to assess these refractive changes [2]. Initial management is conservative and includes lubricating eye drops and protective eyewear to reduce ultraviolet exposure. Surgical intervention becomes necessary when the lesion progresses beyond 3 mm onto the cornea, produces visually significant astigmatism, causes recurrent inflammation, or results in cosmetic disfigurement [3].

Conjunctival autograft transplantation, popularized in 1985, is currently regarded as the preferred surgical technique for reducing recurrence following pterygium excision [4]. This procedure involves placing a free graft of superior bulbar conjunctiva over the bare sclera after removal of the pterygium. Despite its widespread acceptance, recurrence remains a major concern, with reported rates ranging from 2% to 39% in randomized controlled trials. Although conjunctival autografting is widely practiced in centers with advanced microsurgical facilities, its use remains limited in many parts of the world [5].

Several alternative techniques have been introduced over time, including simple conjunctival closure, conjunctival or conjunctivo-limbal autografting, and amniotic membrane transplantation, often combined with adjuvants such as mitomycin C, anti-vascular endothelial growth factor agents, and beta-irradiation. While these approaches have demonstrated reduced recurrence rates, they are associated with their own complications [5].

Limbal-conjunctival autograft transplantation has emerged as a promising modality, particularly for recurrent pterygium. The limbal zone, approximately 0.5 mm wide, lies at the junction of the cornea and sclera [6]. Recognition of limbal stem cells as essential for maintaining corneal epithelial integrity has significantly advanced the understanding of ocular surface disorders. Several studies suggest that limbal stem cell dysfunction contributes to the pathogenesis of pterygium [7]. Limbal autograft transplantation following pterygium excision offers theoretical advantages by restoring limbal architecture and promoting corneal epithelial healing [4]. The limbal epithelium serves as a transitional zone between conjunctival and corneal epithelium, and Tseng proposed that stem cells responsible for corneal epithelial regeneration reside in the basal layer of the limbal epithelium [8]. Limbal autografts have also been shown to facilitate epithelialization in various ocular surface disorders, including corneal burns [7].

The present study aims to compare the outcomes of pterygium excision performed with and without Tenon’s layer excision when combined with conjunctivo-limbal autograft transplantation.

## Materials and methods

Study design and sample size

This prospective comparative study was conducted in the Department of Ophthalmology, Maharishi Markandeshwar Medical College and Hospital, Solan. The sample size was calculated based on previously published studies evaluating recurrence rates following pterygium surgery with and without Tenon’s layer excision [9]. Assuming a minimum detectable difference of 10% in recurrence rates between the two groups, with a confidence level of 95% and a statistical power of 80%, the minimum required sample size was estimated to be 27 patients per group. To account for possible loss to follow-up, 30 patients were included in each group.

Study groups

A total of 60 patients diagnosed with primary pterygium were enrolled over a period of 1.5 years from July 2022 to December 2023. Patients were alternately allocated into two equal groups of 30 patients each. Patients were assigned alternately to one of the following treatment groups: Group A: Pterygium excision with extended conjunctival excision and Tenon’s layer removal, followed by autologous serum-assisted conjunctivo-limbal autograft transplantation. Group B: Pterygium excision with limited Tenon’s layer removal followed by autologous serum-assisted conjunctivo-limbal autograft transplantation.

Selection criteria

Patients with Grade 2 or Grade 3 primary pterygium presenting with visual disturbance, photophobia, watering, foreign body sensation, or cosmetic concerns were included in the study. Pterygium grading was performed based on corneal encroachment as follows: Grade 2 pterygium extended 2-4 mm onto the cornea without involvement of the visual axis, while Grade 3 pterygium extended more than 4 mm onto the cornea and was associated with significant visual disturbance or proximity to the visual axis. Patients with a history of prior ocular surgery, uncontrolled diabetes mellitus, strabismus, or immunocompromised status were excluded.

Preoperative evaluation

All patients underwent a comprehensive ocular examination, including uncorrected and best-corrected visual acuity assessment, slit-lamp biomicroscopy, and keratometry. Relevant clinical findings were documented preoperatively.

Surgical technique

All surgeries were performed by a single experienced surgeon (S.K.) under an operating microscope using peribulbar anesthesia with 2% lignocaine and hyaluronidase. Following sterile preparation and draping, a lid speculum was applied to expose the surgical field. The size of the pterygium was measured using calipers, and the head of the pterygium was separated from the cornea using a combination of blunt and sharp dissection. Subconjunctival fibrovascular tissue, along with the Tenon’s capsule, was excised up to the caruncle, with approximately 5 mm of Tenon’s tissue removed beneath the conjunctival edges. During Tenon’s dissection, care was taken to remain superficial and anterior to the medial rectus insertion. Blunt dissection was preferred near the caruncle, excessive posterior traction was avoided, and dissection was terminated upon identification of resistance suggestive of deeper tissues, thereby minimizing the risk of medial rectus muscle injury. A conjunctival graft was harvested from the supratemporal bulbar conjunctiva and tailored to match the size of the bare sclera, ensuring meticulous dissection to avoid inclusion of Tenon’s tissue. In both groups, conjunctivo-limbal autografts containing approximately 0.5 mm of limbal tissue, including the palisades of Vogt, were transplanted. The graft was positioned with the limbal edge oriented toward the host limbus and recessed by 0.5-1 mm. After avulsion of the pterygium head, residual corneal tissue was gently debrided using a crescent blade to achieve a smooth corneal surface. Mechanical polishing devices, such as an Algerbrush, were not used in this study. Autologous serum was used to secure the graft in place. Autologous serum was obtained intraoperatively from the patient’s peripheral blood collected under sterile conditions. After allowing clot formation, the serum was separated and immediately used to secure the conjunctivo-limbal autograft without the use of sutures or fibrin glue.

Postoperative care and follow-up

Postoperatively, patients received topical moxifloxacin 0.5% eye drops four times daily for one week, and topical prednisolone acetate 1% eye drops four times daily, which were tapered over four weeks based on clinical response. Follow-up examinations were conducted at 24 hours, one week, one month, three months, and six months. At each visit, visual acuity, keratometric readings, and refraction were recorded, and patients were evaluated for graft-related complications, healing, and recurrence.

Statistical analysis

Statistical analysis was performed using IBM Corp. Released 2021. IBM SPSS Statistics for Windows, Version 27. Armonk, NY: IBM Corp. Categorical variables were expressed as frequency and percentage and compared using the chi-square test or Fisher’s exact test, as appropriate. Continuous variables were expressed as mean ± standard deviation and compared using the independent samples t-test or paired t-test. The correlation between pterygium size and astigmatism was assessed using Pearson’s correlation coefficient. A p-value <0.05 was considered statistically significant.

## Results

A total of 60 patients were included in the study, with 30 patients each in the extensive Tenon removal group and the limited Tenon removal group. Most patients were aged 51-60 years (29, 48.3%), followed by 41-50 years (15, 25.0%), 61-70 years (11, 18.3%), and ≤40 years (5, 8.3%). Age distribution was comparable between the two groups (p = 0.942). Males constituted the majority of the study population (43, 71.7%), while females accounted for 17 patients (28.3%), with no significant difference in gender distribution between groups (p = 0.774). All patients had nasal pterygium. Grade 2 pterygium was the most common presentation, observed in 59 patients (98.3%), while Grade 3 pterygium was noted in one patient (1.7%). No patient had Grade 1 pterygium. The distribution of pterygium grades was similar between the two groups, with no statistically significant difference (p = 0.313), indicating comparable baseline clinical characteristics (Table 1).

**Table 1:** Distribution of patients according to demographic and clinical characteristics (chi-square test).

Study characteristic	Category	Excision with extensive Tenon removal n (%)	Excision with limited Tenon removal n (%)	Total n (%)	χ²	p-value
Age (years)	≤40	2 (6.7)	3 (10.0)	5 (8.3)	0.39	0.942
	41–50	7 (23.3)	8 (26.7)	15 (25.0)		
	51–60	15 (50.0)	14 (46.7)	29 (48.3)		
	61–70	6 (20.0)	5 (16.7)	11 (18.3)		
Gender	Female	9 (30.0)	8 (26.7)	17 (28.3)	0.082	0.774
	Male	21 (70.0)	22 (73.3)	43 (71.7)		
Pterygium grade	Grade 1	0	0	0	1.017	0.313
	Grade 2	29 (96.7)	30 (100.0)	59 (98.3)		
	Grade 3	1 (3.3)	0	1 (1.7)		

Mean corneal astigmatism decreased significantly from 2.02 ± 1.10 diopters preoperatively to 0.81 ± 0.54 diopters at six months postoperatively (p < 0.001), indicating a significant reduction in astigmatism following pterygium excision surgery (Table 2).

**Table 2:** Comparison of mean corneal astigmatism before surgery and at 6-month follow-up in all patients (n = 60) (paired t-test).

Time point	Mean astigmatism (D) ± SD	t-value	p-value
Preoperative	2.02 ± 1.10	12.96	<0.001
6 months postoperative	0.81 ± 0.54		

Preoperatively, mean best-corrected visual acuity (BCVA) was 0.05 ± 0.08 logMAR in the extensive Tenon removal group and 0.04 ± 0.08 logMAR in the limited Tenon removal group, with no statistically significant difference between the groups (p = 0.698). At six months postoperatively, mean BCVA improved to 0.01 ± 0.03 logMAR in the extensive Tenon removal group and 0.00 ± 0.00 logMAR in the limited Tenon removal group; however, the intergroup difference remained statistically insignificant (p = 0.321). Overall, both groups demonstrated improvement in BCVA following surgery, with comparable visual outcomes (Table 3).

**Table 3:** Comparison of best-corrected visual acuity (logMAR) between groups before and after surgery (independent samples t-test).

Time point	Treatment group	Mean BCVA (logMAR) ± SD	t-value	p-value
Preoperative	Excision with extensive Tenon removal	0.05 ± 0.08	0.390	0.698
	Excision with limited Tenon removal	0.04 ± 0.08		
Postoperative (6 months)	Excision with extensive Tenon removal	0.01 ± 0.03	1.000	0.321
	Excision with limited Tenon removal	0.00 ± 0.00		

On postoperative day one, subconjunctival hemorrhage was observed in all patients, predominantly mild in nature, occurring in 23 patients (76.7%) in the extensive Tenon removal group and 22 patients (73.3%) in the limited Tenon removal group, with no severe cases reported and no significant intergroup difference (p = 0.766). SCH was graded clinically as mild (limited to the graft margins), moderate (involving more than one quadrant), or severe (diffuse hemorrhage involving most of the bulbar conjunctiva), based on slit-lamp examination. This grading was based on clinical judgment rather than a validated external scale. Squint was absent in all patients (60, 100%). Graft edema was noted in three patients (10.0%) in the extensive Tenon removal group and six patients (20.0%) in the limited Tenon removal group, with the difference not reaching statistical significance (p = 0.278). Graft loss was rare and comparable between groups, occurring in 1 patient (3.3%) in each group (p = 1.000). Graft retraction was observed in two patients (6.7%) in the extensive Tenon removal group and three patients (10.0%) in the limited Tenon removal group (p = 0.640). Graft sliding was seen only in the limited Tenon removal group in two patients (6.7%) and was absent in the extensive Tenon removal group; however, this difference was not statistically significant (p = 0.150). Overall, early postoperative complications were mild and comparable between the two groups (Table 4).

**Table 4:** Comparison of early postoperative complications (day one) between the two groups (chi-square test).

Complication	Category	Excision with extensive Tenon removal n (%)	Excision with limited Tenon removal n (%)	Total n (%)	χ²	p-value
Subconjunctival hemorrhage (SCH)	Mild	23 (76.7)	22 (73.3)	45 (75.0)	0.089	0.766
	Moderate	7 (23.3)	8 (26.7)	15 (25.0)		
	Severe	0	0	0		
Squint	Present	0	0	0	-	-
Graft edema	Present	3 (10.0)	6 (20.0)	9 (15.0)	1.176	0.278
Graft loss	Present	1 (3.3)	1 (3.3)	2 (3.3)	0	1.000
Graft retraction	Present	2 (6.7)	3 (10.0)	5 (8.3)	0.218	0.640
Graft sliding	Present	0	2 (6.7)	2 (3.3)	2.069	0.150

At six-month follow-up, no cases of granuloma, corneal melt, scleral perforation, or dellen were observed in either group (60, 100%). Recurrence occurred in one patient (3.3%) in the extensive Tenon removal group and in four patients (13.3%) in the limited Tenon removal group. Although recurrence was numerically higher in the limited Tenon removal group, the difference was not statistically significant (p = 0.161). Overall, both surgical techniques demonstrated low late postoperative complication rates with comparable safety profiles (Table 5).

**Table 5:** Comparison of late postoperative complications and recurrence at six months between the two groups (chi-square test).

Complication	Category	Excision with extensive Tenon removal n (%)	Excision with limited Tenon removal n (%)	Total n (%)	χ²	p-value
Granuloma	Present	0	0	0	–	–
Corneal melt	Present	0	0	0	–	–
Scleral perforation	Present	0	0	0	–	–
Dellen	Present	0	0	0	–	–
Recurrence	Present	1 (3.3)	4 (13.3)	5 (8.3)	1.964	0.161

Pearson’s correlation analysis demonstrated a mild positive correlation between pterygium size and corneal astigmatism (r = 0.30), which was statistically significant (p = 0.016), indicating that larger pterygia were associated with higher degrees of corneal astigmatism (Table 6).

**Table 6:** Correlation between pterygium size and corneal astigmatism.

Variables compared	Pearson’s correlation coefficient (r)	p-value
Pterygium size vs. corneal astigmatism	0.30	0.016

**Figure 1:**
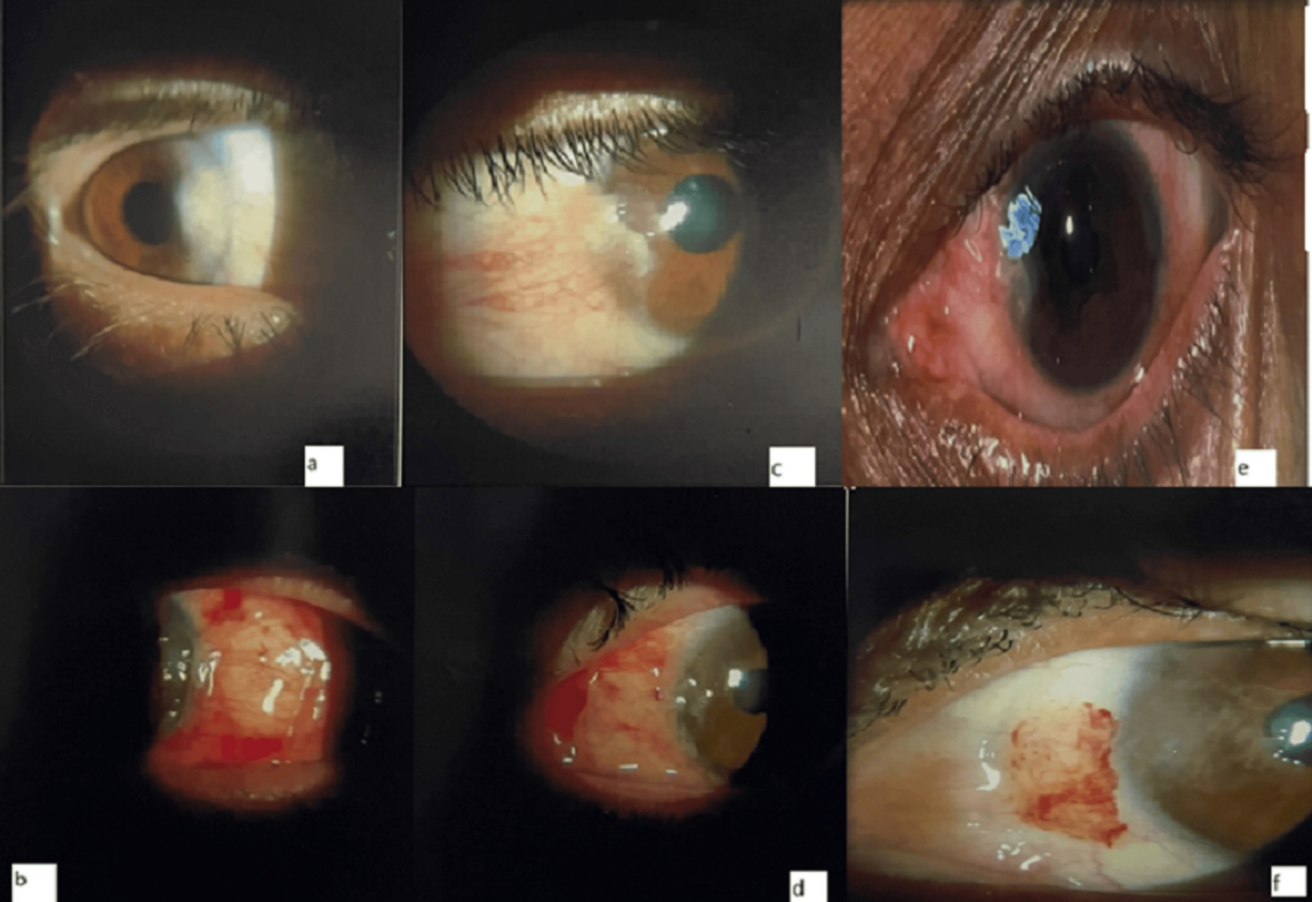
Slit-lamp photographs of a 55-year-old male patient from the extended Tenon removal group showing preoperative and postoperative findings. (a) Preoperative appearance of nasal pterygium encroaching onto the cornea. (b) Immediate postoperative appearance following pterygium excision showing bare sclera. (c) Preoperative pterygium with associated conjunctival congestion and corneal involvement. (d) Early postoperative appearance after conjunctivo-limbal autograft placement. (e) Well-positioned conjunctivo-limbal autograft at follow-up with satisfactory graft adherence. (f) Healed ocular surface at late postoperative follow-up without evidence of recurrence.

Slit-lamp examination demonstrated progressive resolution of conjunctival congestion and restoration of ocular surface integrity following pterygium excision with conjunctivo-limbal autograft transplantation. Early postoperative images showed stable graft placement with satisfactory adherence, while late postoperative images revealed a well-epithelialized ocular surface without signs of graft displacement or recurrence (Figure 1).

## Discussion

Pterygium surgery remains the definitive treatment for improving visual acuity by reducing corneal astigmatism and removing fibrovascular tissue from the visual axis [3]. The present study compared outcomes of conjunctival excision with extended Tenon’s layer removal and autologous serum-assisted conjunctivo-limbal autograft transplantation with the same procedure performed with limited Tenon removal.

The mean age of patients in the present study was 53.45 ± 8.42 years, with the majority belonging to the 51-60-year age group. No significant difference in age distribution was observed between the two groups, which is comparable with findings reported by Garg et al., Das et al., and Wiącek et al. [10-12]. A male predominance (71.7%) was observed, consistent with earlier studies [10,12,13], likely reflecting greater occupational and environmental exposure to ultraviolet radiation. Laterality was nearly equal, and all cases involved nasal pterygium, similar to observations by Chauhan et al. and Das et al. [11,13]. Most patients had Grade 2 pterygium, with no significant difference in grading between groups, comparable to previously reported data [11,13,14].

Preoperatively, most patients had corneal astigmatism between 2.1 and 3.0 diopters. At six-month follow-up, a significant reduction in astigmatism was observed, with the majority of patients demonstrating less than 1 diopter of astigmatism. The mean astigmatism decreased significantly from 2.02 ± 1.10 D preoperatively to 0.81 ± 0.54 D postoperatively (p < 0.05). Both surgical techniques produced comparable reductions in astigmatism, with no statistically significant intergroup difference. These findings are consistent with reports by Garg et al. [10], Mohite et al. [15], and Cinal et al. [16], who demonstrated significant postoperative improvement in corneal astigmatism following pterygium excision.

The reduction in corneal astigmatism after surgery is likely attributable to restoration of corneal surface regularity and reduction of fibrovascular traction on the cornea [17]. In the present study, a mild positive correlation was observed between pterygium size and the degree of astigmatism (r = 0.30, p = 0.016), indicating that increasing lesion size is associated with greater refractive distortion. Similar correlations have been reported by Gumus et al. and Seitz et al., who demonstrated that larger pterygia induce greater corneal astigmatism [18,19].

Although no significant difference in best-corrected visual acuity (BCVA) was observed between the two groups, overall visual acuity improved significantly following surgery. Mean logMAR BCVA improved from 0.04 ± 0.08 preoperatively to 0.00 ± 0.02 at six months. These findings are consistent with previous studies demonstrating improvement in visual acuity following pterygium excision [2,10,20]. Improvement in vision is largely attributed to a reduction in corneal astigmatism and removal of tissue obstructing the visual axis.

Barraquer proposed that excision of Tenon’s layer plays an important role in reducing recurrence after pterygium surgery [21]. Solomon et al. further emphasized this concept, achieving low recurrence rates using extensive Tenon excision combined with adjunctive therapies [22]. In the present study, early postoperative complications such as subconjunctival hemorrhage, graft edema, graft retraction, and graft loss were comparable between groups. Graft sliding was observed only in the limited Tenon removal group. Although recurrence was lower in the extended Tenon removal group (3.3% vs. 13.3%), this difference did not reach statistical significance, possibly due to the limited sample size and follow-up duration.

The importance of Tenon’s layer excision has also been highlighted by Ciftci et al., who demonstrated reduced recurrence when Tenon’s tissue was thoroughly removed [9]. Hirst reported near-zero recurrence rates following extensive Tenon excision; however, his technique involves aggressive tissue manipulation and carries a higher risk of complications related to extraocular muscle pulley disruption [23]. Interestingly, Hirst also suggested that limbal stem cell transplantation may not be essential for achieving low recurrence rates if Tenon’s tissue is adequately excised [24].

The present study uniquely evaluates the influence of Tenon’s layer in the presence of conjunctivo-limbal autografts. The findings suggest that Tenon’s layer plays a more substantial role in recurrence than limbal stem cell transplantation alone. The high success rates reported with conjunctival autografts and limbal-conjunctival autografts may largely be attributed to adequate Tenon excision rather than limbal stem cell replacement alone [25]. Recent studies have reinforced that meticulous Tenon’s layer excision plays a critical role in reducing recurrence, sometimes outweighing the contribution of limbal stem cell transfer alone. Contemporary comparative studies have demonstrated that conjunctival or limbal-conjunctival autografting with adequate Tenon removal yields recurrence rates comparable to mitomycin C-augmented procedures, while avoiding the potential sight-threatening complications associated with antimetabolite use [23,26,27].

Several randomized and comparative studies published in the last two decades have evaluated mitomycin C as an adjunct in primary pterygium surgery, reporting low recurrence rates but with concerns regarding scleral thinning, delayed epithelial healing, and corneal complications, thereby supporting safer tissue-based approaches when feasible [5,26].

Overall, pterygium appears to be a multifactorial disorder, and no single surgical modification is sufficient to eliminate recurrence entirely. The present study supports a comprehensive surgical approach that combines conjunctivo-limbal autograft transplantation with thorough Tenon’s layer removal to optimize outcomes and reduce recurrence risk.

This study has certain limitations. The sample size was relatively small, which may have limited the ability to detect statistically significant differences in recurrence rates between the two groups. The follow-up period of six months may not fully capture late recurrences, which are known to occur beyond this duration. Additionally, alternate allocation rather than true randomization may introduce selection bias. Future studies with larger sample sizes, longer follow-up, and randomized controlled designs are warranted to validate these findings.

## Conclusions

This study demonstrates that conjunctivo-limbal autograft transplantation combined with extended Tenon’s layer removal is associated with a lower recurrence rate and fewer graft-related complications compared with limited Tenon removal, although the differences were not statistically significant. Both surgical techniques resulted in significant improvement in visual acuity and reduction in corneal astigmatism. The findings underscore the contributory role of Tenon’s layer in pterygium recurrence and support a comprehensive surgical approach that includes adequate Tenon excision to optimize outcomes. Further studies with larger sample sizes and longer follow-up are required to confirm these observations and refine surgical strategies.
